# Aerosol emissions and gravity waves of Taal volcano

**DOI:** 10.1038/s41598-022-09281-y

**Published:** 2022-03-28

**Authors:** Jan-Bai Nee, Yuan-Pin Chang, Chia C. Wang

**Affiliations:** 1 Aerosol Science Research Center, National Sun Yat-Sen University, No. 70 Lien-hai Rd., Kaohsiung, 80424 Taiwan, ROC; 2 Department of Chemistry, National Sun Yat-Sen University, No. 70 Lien-hai Rd., Kaohsiung, 80424 Taiwan, ROC

**Keywords:** Environmental sciences, Physics

## Abstract

The Taal volcano (14.0 N, 121.0 E) in Philippines erupted in January–February 2020, with a part of aerosols drifted northward and detected by a lidar system at Kaohsiung city (22.37 N, 120.15 E), Taiwan. The aerosol observed on Feb 11 is special for its high-altitude distributions at 4–7 km with discrete structures which can be resolved into a sinusoidal oscillation of ~ 30 min period, suggesting a case of wave event caused by the eruptions. We report in this paper the gravity wave generated by the volcanic eruptions and its effects on aerosol emissions. By studying the temperature and pressure data in the Taal region using radiosonde data, we found atmospheric gravity waves with powers correlated with the optical thickness (AOD) at 550 nm measured by the Moderate Resolution Imaging Spectrometer (MODIS) satellite. This study presents the first observation of modulation of the aerosol emissions by the volcanic gravity waves and a case of coupling of dynamics and chemistry.

## Introduction

Volcanic eruptions can significantly affect the environment and climate by sending large amounts of aerosols and gases into the atmosphere^1^. For example, the recent eruption of Iceland volcano Eyjafjallajökull has severely impaired the air quality and disturbed the air traffic in Europe for a few months. Additionally, volcano eruptions can also disturb the atmosphere by producing infrasound and gravity waves as shown in past eruptions of Pinatubo, Mt. St.Helen, and El Chichon^2–7^. For these major eruptions, pressure oscillations of amplitudes of a few millibars (mb) and periods about 200–300 s following the initial blasts have been recorded by global instruments^3–7^. Atmospheric waves are generated when the air is perturbed from equilibrium by external forces like volcanic eruption, convection, wind shear and so on. The propagation of these waves may be 3D with vertical height reaching as far as ionosphere^8–10^. Volcanic gravity waves were also observed for moderate eruptions such as the Okmok volcano^5^ and Soufrière Hills Volcano^6^ among others. Although there have been extensive studies of gravity waves and their effects on various parts of atmosphere, little is known about the effects of gravity waves on the volcanic aerosols.

Gravity waves are generated when the stable air is perturbed by external agents such as convection, wind shear, or volcano eruptions. Following the perturbation, the atmosphere will oscillate because of the counteraction of gravity force to restore the displacement to its stable status similar to a box-spring oscillation. Dynamics about the volcanic gravity waves has been investigated by several authors^4–8^. In the past, the atmospheric gravity waves are mostly observed in atmospheric parameters, such as pressure, temperature, wind field, humidity and so on^3,8^. A few studies addressing the gravity wave-related effects on aerosols have been reported^11–13^. However, gravity waves discussed in these papers are formed by wind shear or stratospheric dynamics and are unrelated with volcanic gravity waves. Recently, Baines and Sacks^13^ modelled the generation of stratospheric gravity waves by volcano eruptions but not consider their effects on aerosols. Klekociuk et al.^12^ reported the displacement of stratospheric aerosols by passing gravity waves of atmospheric origin. In general, the volcanic gravity waves have been extensively studied but mostly on their effects on seismic activities and upper atmospheric air^3–13^ while their effects on aerosols is little explored.

The present study is motivated by the observations of a high lying aerosol layer at 4–7 km with a discrete structure measured by an aerosol lidar at Kaohsiung city (KS) which is about 1000 km north of Taal. This aerosol layer is special compared to normal aerosols of pollution types which are often distributed at heights below 3 km with continuous structure in time. By using back-trajectory calculations, we find this aerosol was sourced in the Taal region. To better understand this aerosol event, we investigated the eruptions of Taal volcano by combining ground and space-based data. The paper is written in the following order: the next section is about the background information of the Taal volcano and data systems used in this study which include MODIS and Merra-2 (Modern-Era Retrospective Analysis for Research and Applications, Version 2), the HYSPLIT (Hybrid Single-Particle Lagrangian Integrated Trajectory) trajectory calculations, and the lidar system. The third section is the data analysis including both the lidar and atmospheric data in the source region. The fourth part is the discussion of gravity waves related to the Taal volcano and the effects on aerosols. The last part is the conclusion of this paper.

## Background information

### Taal volcano

Taal volcano (14 N,121 E) is located at Luzon Island of Philippines about 60 km south of Manila. Taal is the second most active volcanos in the Philippines with 33 historical eruptions since AD1572 with the largest eruption in AD1754 rated as volcano index (VEI) 5, compared with Pinatubo in 1991 of VEI 6^14^. Starting Jan. 12, 2020, the volcano erupted again producing plumes of hot gases, lightnings, and tephra (volcanic materials) at heights of 10–15 km according to the report of Philippine Institute of Volcanology and Seismology (PHIVOLCS) as shown in Global Volcanism Program^15^.

**Figure 1:**
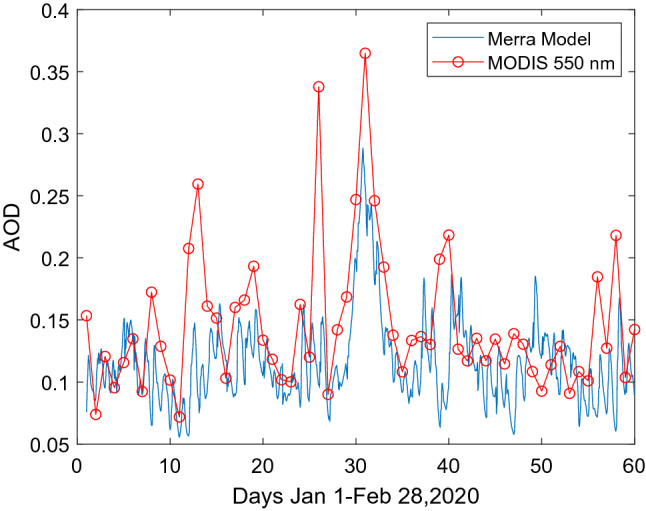
Merra-2 Model aerosol extinction at 550 nm and MODIS deep blue time series, area-averaged AOT 550 nm over Taals region (in areas of Longitudes:119°–123°, Latitudes 12°–16°).

The 2020 eruption is investigated by using the area-averaged aerosol optical depths (AOD) at 550 nm over the Taal region as shown in Fig. 1 which is based on MODIS data and Merra-2 model. The AOD 550 nm is observed to start to rise in mid-January with the largest AOD in the end of January to early February and a few peaks in various days. More detailed introduction of MODIS and Merray-2 model are given in the following sub-sections.

### MODIS satellite

The MODIS data in Jan.–Feb. 2020 are downloaded to study the aerosol optical depth over Taal region. MODIS data are daily measurements based on observations of Terra and Aqua satellites launched by NASA in 1999 and 2002, respectively. Both satellites observe Earth using 36 bands of wavelengths ranging from 0.41 to 15 μm. This spectral diversity allows MODIS to retrieve aerosol optical parameters characterizing aerosol sizes and properties^16–19^. The deep blue products used in this study are designed for bright-reflecting land surfaces, such as desert, semiarid, and urban regions^19^. The MODIS aerosol optical depths (AOD) at 550 nm can be downloaded from the NASA Giovanni website.

### Merra-2 model

Since most satellite observations are daily measurements based on their orbit design, the data are discrete in time. For example, Terra MODIS and Aqua MODIS are viewing the entire Earth's surface every 1 to 2 days so that the dataset are with spatial and temporal discontinuities. In general satellite measurements are also affected by cloud interferences causing breaks in cloudy days. The Merra-2 is the reanalyses of atmospheric data by combining model fields with observations of irregular space and time into a spatially complete gridded dataset. Reanalysis means a scientific method for developing a comprehensive record of atmospheric parameters. Currently, the MERRA-2 dataset spans the period 1979 through February 2016 producing data on a geographically 0.5° × 0.66° grid with 72 layers. In this work, we have used the time-series, area-averaged total aerosol scattering AOT 550 nm at Taal region. For aerosol research, MODIS provides the vast majority of AOD observations assimilated in MERRA-2^20^. For more information, please visit the NASA MERRA-2 website.

### HYSPLIT system

HYSPLIT, developed by ARL (Air Resources Laboratory, NOAA), is designed to compute sample air parcel trajectories, transport, dispersion, chemical transformation, and deposition simulations^21,22^. Fleming et al.^21^ presented a review about the development of the HYSPLIT systems for understanding the influence of air-mass history of atmospheric pollution parcels and to examine the source–receptor relationships in the timescales of long- range transport. This model involves a hybrid calculation between the Lagrangian approach and the Eulerian approach. The former uses a moving frame of reference for the traveling air parcels, the later used a fixed 3D frame to compute the paths of moving pollution.

**Figure 2:**
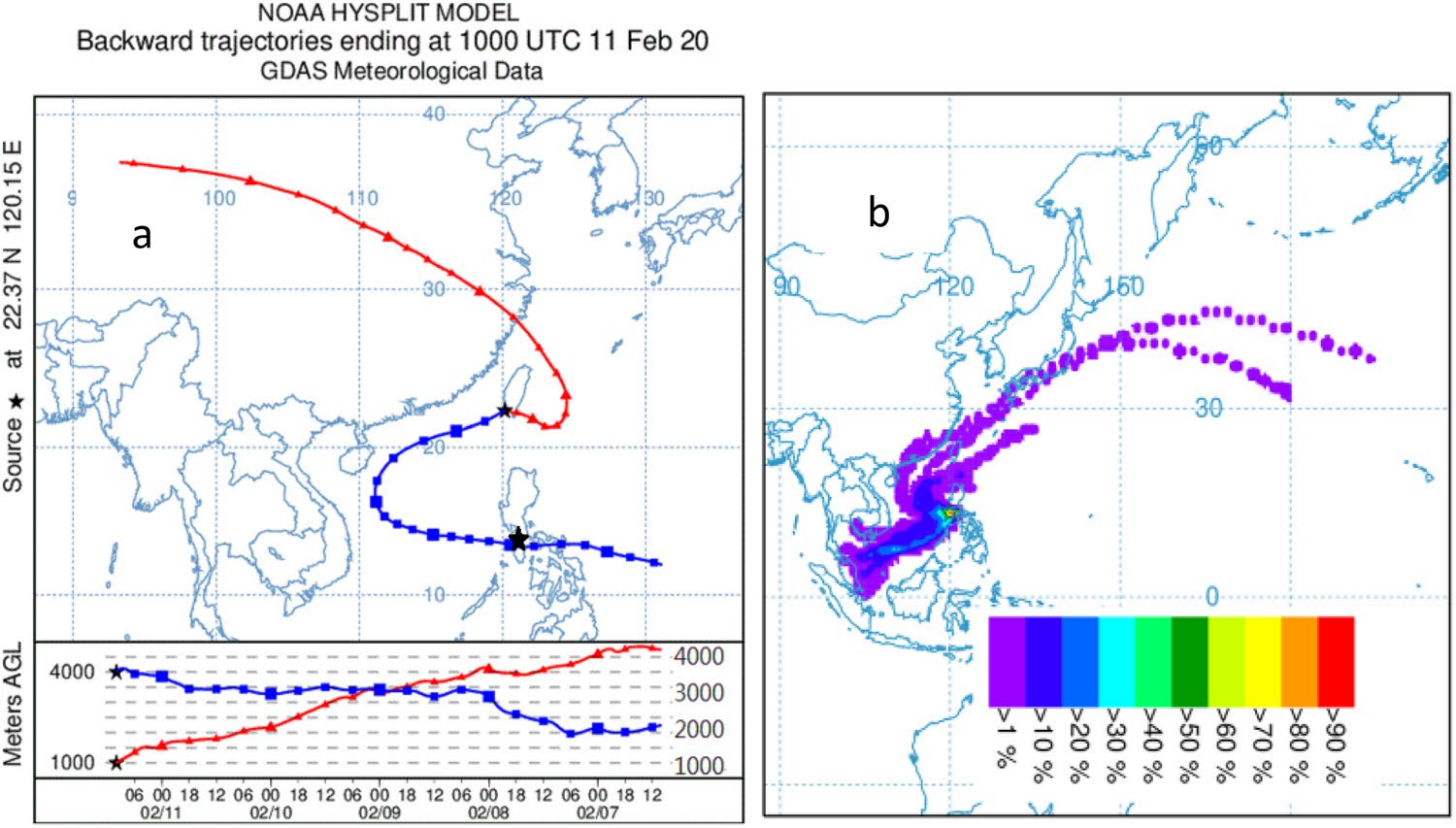
(**a**) Five-day back-trajectories for aerosols at 1 km and 4 km layer, both were observed by lidar at Kaohsiung. The 4 km layer (blue) can be trace to Taal region 3 days ago on Feb. 8 at 2000 m while the 1 km layer (red) is traced to northwestern China from upper air. (**b**) forward trajectories from Taal region starting Feb.8 (based on backward study in **a**) for all possible transport paths in the next 5 days. The results show a small part of aerosols may move to north in KS while the majority went to Southeast Asia. Figures are plotted using HYSPLIT Trajectory Model provided by ARL NOAA^21,22^.

The back-trajectory calculations in this paper are carried out by using the GDAS model (Global Data Assimilation System) with one-degree resolution (latitude-longitude) to calculate the paths of Kaohsiung air at 1000 m and 4000 m heights in the past 5 days since aerosols at both layers were observed by the lidar system. The 1000 m layer is found to be a continental aerosol transported by the Asian Northeast Monsoon system in the wintertime with sources often found in the Taklamakan or Gobi deserts. This aerosol is not further studied. Aerosols at 4000 m, at the base height in the lidar measurements, are traced to Taal region at about 2000 m heights as shown in Fig. 2a. They were raised to 4000–7000 m heights arriving KS, Taiwan. Taal is located within the ITCZ (Inter-Tropical Convergence Zone) of ± 15° latitudes where predominant rising air exists due to strong convective systems in the tropics. Figure 2b shows the forward trajectories starting from Taal region on Feb. 8. Based on backward studies, aerosols on Feb. 8 in the source region transported to KS, Taiwan. In order to confirm this, all forward transport paths for aerosols in the next 5 days starting Feb. 8 are calculated. Forward study shows aerosols from Taal region can reach to Taiwan area although most of them were transported to the Southeast Asia. A combination of backward and forward trajectories allows better understandings about the source of aerosols.

### Lidar systems and measurements

**Figure 3:**
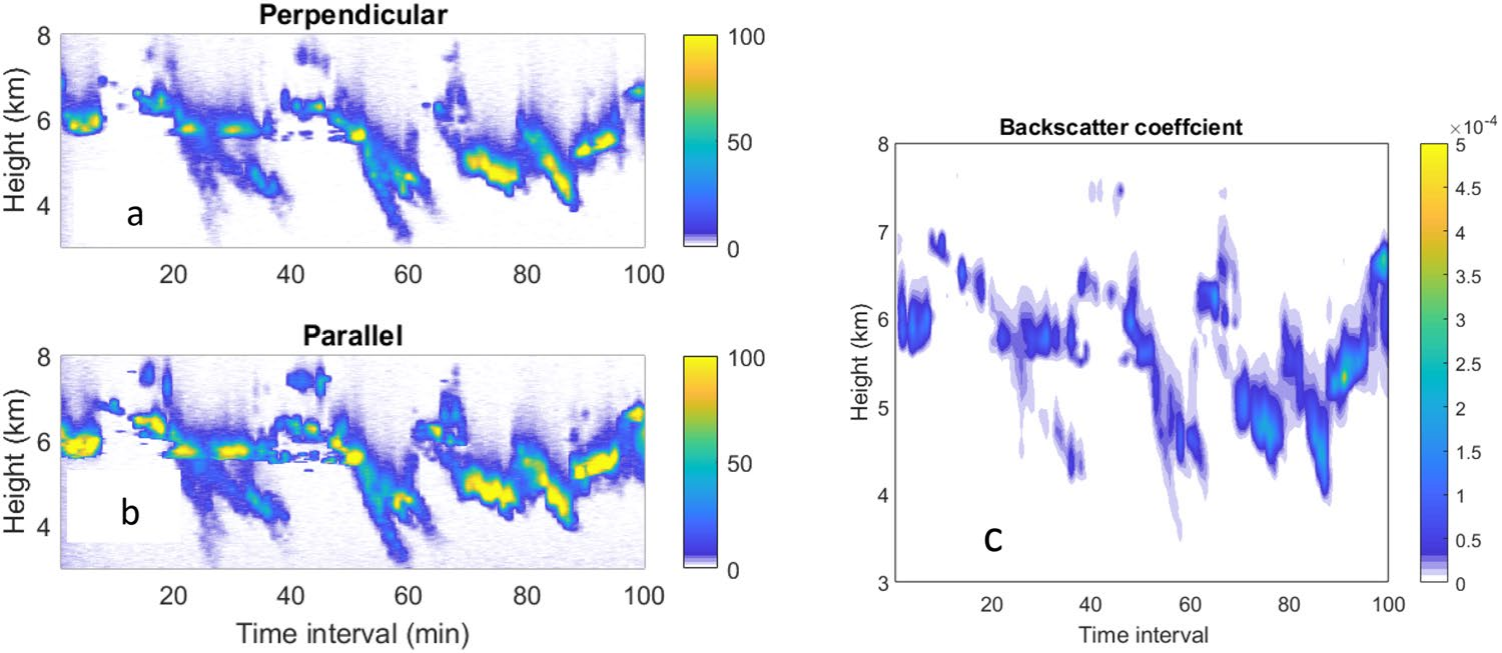
(left) Aerosol layers measured by lidar at Kaohsiung in signals of perpendicular (**a**) and parallel (**b**) polarization channels, time starting 18:04 LT. (**c**)The backscattering coefficients in unit of (1/m-sr) measured on Feb 11.

The lidar system used in this study is located in the campus of National Sun Yat-sen University in Kaohsiung city (22.37 N, 120.15 E). It has been setup to observe aerosols routinely since 2019. The lidar system consists of a laser transmitter using the 532 nm laser wavelength and a receiver of a 20 cm diameter Cassegrain telescope (Celestron 8) as shown in our previous publication^23^. Laser light sending to the sky will continually interact with the atmosphere with the backscattering signals collected by the telescope. Lidar signals are measured by using a combination of photomultiplier tube (Hamamatsu 9880–10), interference filters, and polarization filters. Signals are analyzed by a transient digitizer (Licel system) to generate profiles of signals with a height resolution of 3.75 m. The polarization dependent backscattering signals detected in parallel and perpendicular directions relative to the polarization of the laser emission are shown in Fig. 3a,b. Figure 3c displays the backscattering coefficients (1/m-sr) derived by an inversion mechanism which has been reported previously^23^.

## Analyses and results

### Optical properties of Taal aerosols

**Figure 4:**
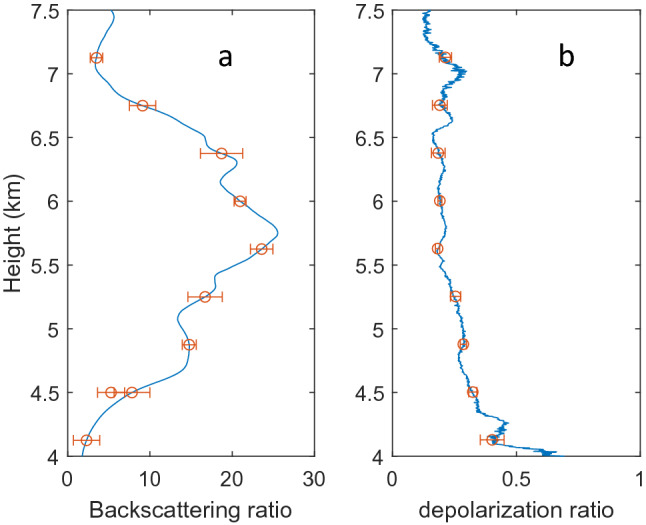
(**a**,left) Time series of integrated signals in 4.5–6.00 km heights, (**b**,right) FFT power of signals.

The optical properties of aerosols observed by lidar are discussed here. The backscattering coefficients shown in Fig. 3c are used to derive the backscattering ratios (BR) shown in Fig. 4a. BR is defined as ratio of backscattering coefficient relative to that of the Rayleigh scattering of air. From Fig. 4a, we find the peak BR is at 5.78 km. The mean optical depth based on the lidar data is 0.22 ± 0.26 which may be compared with Merra-2 and MODIS AOD at 550 nm shown in Fig. 1. The depolarization ratio (DR), defined as the ratio of signals of perpendicular to parallel polarizations, is a parameter for describing the shape of particles, with zero DR for round particles such as liquid droplets of stratospheric aerosols^1^. For the Taal case, we find a DR with a mean of 0.251 ± 0.096 at heights of 4–7.5 km as shown in Fig. 4b, corresponding irregular shaped ashes. Volcanic aerosols are sources of stratospheric aerosols which are in the forms of droplets of sulfuric acid^1^. For the Taal eruption, the aerosols detected are likely small ashes from the eruptions just a few days ago not yet reaching stratosphere. The depolarization ratio of Taal volcano may be compared with that of Eyjafjallajökull which has a depolarization ratio about 0.35 as reported by several groups^23–25^.

### Gravity wave of Taal

**Figure 5:**
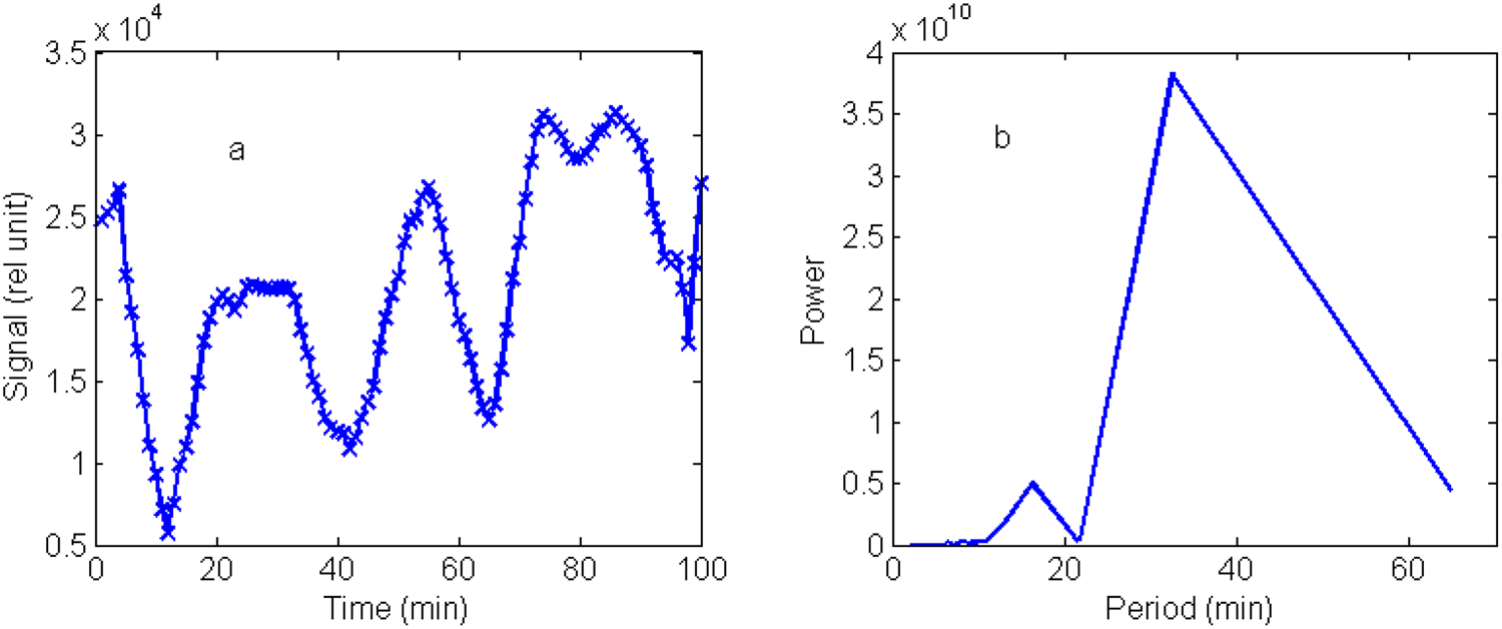
(**a**, left) Time series of integrated signals in 3–8 km heights (**b**, right) FFT power of signal of Fig. (5a) with the major peak at 32.5 min and a minor peak at 16.2 min.

The wave characteristics of aerosols are studied in the following ways. From Fig. 3a,b, we can see the aerosol are mainly distributed over 4–7 km height ranges. The peak-to peak signals over 3–8 km heights are integrated and plotted against time as shown in Fig. 5a in which a sinusoidal oscillation of a period about 30 min lasting for about an hour is observed. The integrated signals are further analyzed by the Fast Fourier Transform (FFT) with the power spectrum showing a major peak period of 32.5 min and a minor one at 16.22 min in Fig. 5b. The secondary peak is half of the period, or double frequency, of the major peak. We attribute this oscillation to the perturbation of aerosols by gravity waves. It is likely that the eruption of the Taal volcano perturbed the emissions of aerosols, producing discrete distributions of aerosol parcels that eventually transported to distant regions as observed by the lidar system in KS.

**Figure 6:**
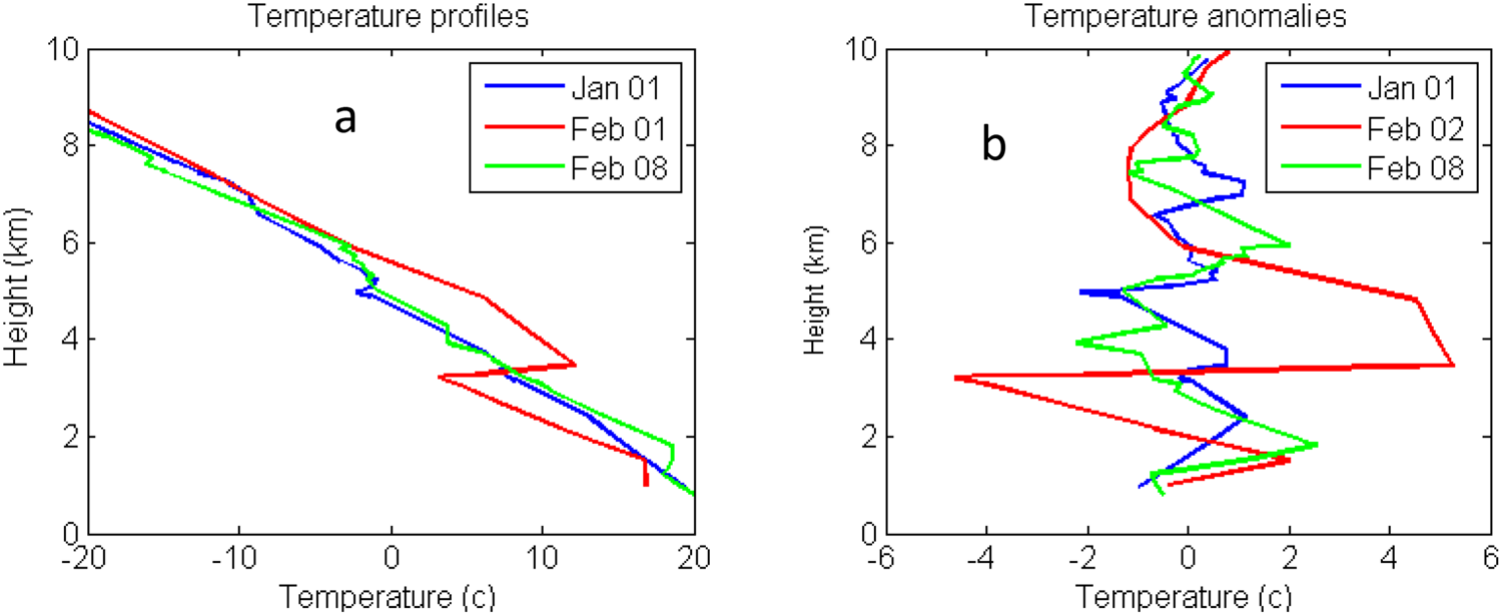
(**a**) Temperature profiles of Taal region on Jan 1, Feb 1, and Feb 8; (**b**) Temperature anomalies.

**Figure 7:**
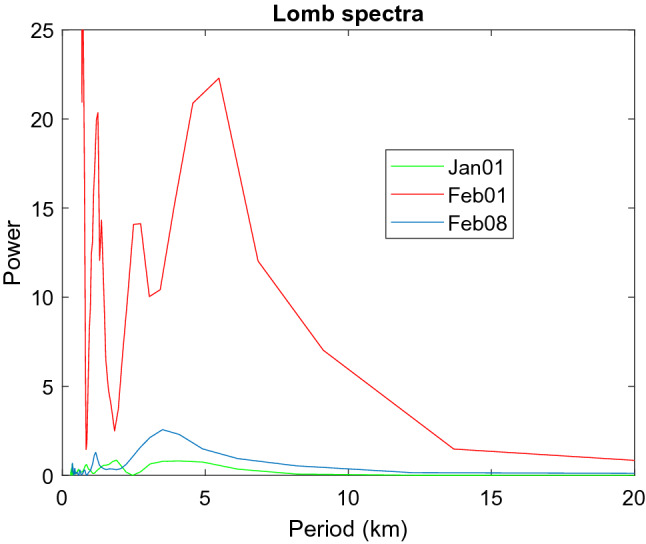
Power spectrum of the Lomb-Scargle analyses for temperature anomaly of Fig. 6b.

To understand the atmospheric conditions in the source region, we have studied the temperature profiles of the Taal region by using the radiosonde data (Station no. 98443, Lat. 14.56, Long. 121.36) for two months of January–February 2020. The temperature data of several days corresponding to before (Jan 1) and during (Feb.1 and 8) the eruptions are shown in Fig. 6a. The variations of temperature (or pressure, not presented here) for these days are investigated in the following ways. We first derived the mean temperature profiles of each day by using a polynomial fitting technique. The temperature perturbations for these days are then calculated by subtracting the mean temperatures from the observed temperature profiles as shown in Fig. 6b. The Lomb–Scargle periodogram (LS) which is suitable for discrete data analysis is applied to find the characteristic frequencies of oscillations using the temperature anomaly in Fig. 6b. The LS results in Fig. 7 show atmospheric waves of vertical wavelengths of 3–5 km in heights of 1–10 km. Results of Fig. 7 is typical for finding gravity wave activities in the atmosphere.

In the power spectra of Fig. 7, the strongest peak appears on Feb.1 whose AOD 550 nm is also the largest as shown in Fig. 1. The smallest power intensity occurs on Jan. 1 corresponding to the day of before the eruption, and a moderate power for Feb. 8 during the eruption. Feb. 8 is the origin of lidar aerosols based on back-trajectory studies discussed earlier. Therefore, the powers of the wave activities correlate well with the optical thickness of the region given by MODIS AOD and Merra-2 model. Similar results are also found for pressure data but with much lower powers and are not shown here. Table 1 lists the relevant parameters discussed above.

**Table 1:** A list of parameters.

Parameters observed	Data
Lidar location	22.37 N, 120.15 E
Source region (Taal)	14.00 N,120.59 E
Aerosol layer height	4–7 km
Time of lidar observation (Feb. 11)	6–8 PM, Feb 11,2020
Period of oscillation	32.5 min
Wind direction 5–6 km (Feb. 8)	330°
Horizontal wind speed (Feb 8)	5 m/s @5 km
Brunt-Vaisala frequency @5 KM	0.022 s^−1^, period 282 s
Gravity wave vertical wavelength	3–5 km
Average aerosol optical thickness	0.22 ± 0.26 at 3–8 km
Average depolarization ratio	0.251 ± 0.096

## Discussion

Here we briefly describe the gravity waves to gain insight into the possible perturbations produced by the Taal eruptions. The displacement of air in a stable atmosphere is explained by the Brunt-Vaisala (BV) frequency, N(z), which is the frequency of air parcel oscillation. The frequency N(z) is calculated by the following formula

1 $${\text{N}}^{{2}} \left( {\text{z}} \right) = \left( {{\text{g}}/{\text{T}}\left( {\text{z}} \right)} \right) \, \left( {{\text{dT}}\left( {\text{z}} \right)/{\text{dz}} + {\text{g}}/{\text{c}}_{{\text{p}}} } \right)$$

where g is the gravity constant 9.8 m/s^2^, c_p_ is heat capacity of air 1004 J/deg-kg, and T(z) is the temperature. N(z) is found to be 0.022 rad s^−1^ at z = 5 km, corresponding to a period about 280 s which is close to ~ 300 s period described in earlier studies about volcanic gravity waves^4^. The BV frequency is the maximum possible frequency for the gravity wave since the wave can propagate both horizontally and vertically^8^. Considering only 2D plane wave for simplicity, the wave function can be written as

2 $${\text{u}} \sim {u^{\prime}}{\exp}({\text{i}}({\text{kx}} + {\text{mz}} - {\omega\text{t}}))$$

where u’ is the perturbation of the atmospheric parameters including temperature, wind speed etc., k and m are the horizontal and vertical wavenumbers, *ω* is the wave frequency. The relationship between frequency and wavenumber is given by the dispersion relation ^2,8^

3 $${\text{m}}^{{2}} = {\text{k}}^{{2}} ({\text{N}}^{{2}} - {\omega}^{{2}} )/({\omega}^{{2}} - {\text{f}}^{{2}} )$$

where N is the Brunt–Vaisala frequency, and f is the Coriolis inertial frequency of the earth rotation at a latitude α defined as f = 2Ωsin α = 3.51 × 10^–5^ s^-1^, Ω is the earth’s rotation speed. For a 2D wave propagating at a directional angle β relative to the horizontal direction, the frequency is:

4 $$\omega = {\text{Ncos}}\beta < {\text{ N}}$$

If there is a background horizontal wind of speed u_o_, the wave frequency is modified by the wind vector u_o_ as^8^

6 $$\omega^{\prime} = \omega - {\text{u}}_{{\text{o}}} {\text{k}}$$

Although the vertical wavelength is a few kilometers as shown in Fig. 7, the horizontal wavelength may be hundreds or thousands of kilometers^8^. The volcanic gravity waves can modulate the aerosols to cause oscillations but with a frequency different from that of air because aerosols are heavier than air. The average aerosol mass is about 1.5 g/cm^3^^24–26^ which is 1000 times or more than the density of air. Using a relationship ω ~ 1/m^-2^ based on box spring oscillation, we find aerosols should have a wave period about 2000s by comparison with the BV frequency of 280 s of air. This estimation is close to the lidar observations but may be fortuitous. The gravity wave parameters discussed above are summarized in Table 1.

From Fig. 3a,b, we found that aerosols are mainly distributed over heights of 4–7 km. This vertical spread should result from the action of oscillations produced by the gravity waves and can be understood in terms of the box-spring model. At the spring’s maximum stretch, aerosols are pushed to the peak height over 7 km and back to the lowest height when the spring is relaxed. Therefore, gravity waves perturbed the distribution of aerosols vertically with a structure related with the oscillation period.

Recently Klekociuk et al.^12^ reported a case of stratospheric aerosols modulated by the gravity wave. A displacement of stratospheric aerosols over a distance of 600 m was observed produced by passing gravity waves. The perturbation will cause aerosols to distribute over different heights or different atmospheric temperatures which can affect the condensation or coagulation of aerosols. In the present case, the spread is much broader and the temperature variation is about 10 degrees which can significantly affect aerosol condensation, coagulation, and cloud formation. Furthermore, variation of the coagulation of aerosols also affects the sedimentation of aerosols that can impact the environment. These effects remain to be further clarified but is beyond the scope of this paper.

## Conclusion

Volcanic aerosols have long been studied to better understand their roles in affecting the environment and climate of Earth. However, volcanic eruptions can also induce dynamic activities of the atmosphere by producing gravity waves which may influence the distributions of gas or aerosol emissions. It is well known that volcanic emissions of CO_2_, H_2_O, and SO_2_, in addition to aerosols, are important in affecting the stratospheric chemistry and greenhouse effects. However, their dynamic effects have not been studied. In this paper, we report a case of a discrete aerosol layer produced by Taal eruption observed by a lidar system at Kaohsiung city, Taiwan, which is 1000 km north to the Taal region. The integrated signals over 3–8 km show a sinusoidal oscillation with a period about 32.5 min. With additional data of MODIS AOD, Merra-2 model, radiosonde temperature, and back-trajectory calculations, we studied the possible perturbation of volcanic aerosols by the volcanic gravity waves. The temperatures profiles from the radiosonde data of the source region are analysed by Lomb-Scargle (LS) periodogram for days before and during the eruptions for understanding the atmospheric dynamics with the power spectra showing correlation with the AOD of these days. These results support the possibility of gravity waves generated by the volcanic eruptions can affect aerosol emissions producing aerosol layers distributed over different heights when transport to distant regions. However, considering the long transport for aerosols to reach the lidar site, we cannot rule out possibilities of the gravity wave activities also caused by the convective clouds or other weather systems in the tropical region where aerosols travelled, although such events have not been observed by us so far. Under either condition, this is a case of modulation of aerosol structures by atmospheric dynamics resulting redistribution of aerosols spatially with the possibility of affecting the coagulation, condensation, sedimentation, and cloud formation. These effects have so far not been systematically explored but is noteworthy for future research in the context of aerosol-climate systems.
